# Functional Fermented Milk with Fruit Pulp Modulates the In Vitro Intestinal Microbiota

**DOI:** 10.3390/foods11244113

**Published:** 2022-12-19

**Authors:** Tais Fernanda Borgonovi, Mateus Kawata Salgaço, Gislane Lelis Vilela de Oliveira, Lucas Amoroso Lopes de Carvalho, Daniel Guariz Pinheiro, Svetoslav Dimitrov Todorov, Kátia Sivieri, Sabrina Neves Casarotti, Ana Lúcia Barretto Penna

**Affiliations:** 1 Graduate Program in Food, Nutrition and Food Engineering, UNESP—São Paulo State University, São José do Rio Preto 15054-000, SP, Brazil; 2 Graduate Program in Agricultural and Livestock Microbiology, UNESP—São Paulo State University, Jaboticabal 14884-900, SP, Brazil; 3 Department of Agricultural, Livestock and Environmental Biotechnology, UNESP—São Paulo State University, Jaboticabal 14884-900, SP, Brazil; 4 ProBacLab, Laboratory of Food Microbiology, Department of Food Science and Experimental Nutrition, Faculty of Pharmaceutical Sciences, University of Sao Paulo, São Paulo 05508-000, SP, Brazil; 5 Department of Food and Nutrition, School of Pharmaceutical Science, UNESP—Sao Paulo State University, Araraquara 14800-903, SP, Brazil; 6 Faculty of Health Sciences, UFR—Federal University of Rondonópolis, Rondonópolis 78736-900, MT, Brazil; 7 Institute of Biosciences, Humanities and Exact Sciences, Department of Food Engineering and Technology, UNESP—São Paulo State University, São José do Rio Preto 15054-000, SP, Brazil

**Keywords:** functional food, intestinal health, microbial interaction, food–gut axis

## Abstract

The effect of putative probiotic fermented milk (FM) with buriti pulp (FMB) or passion fruit pulp (FMPF) or without fruit pulp (FMC) on the microbiota of healthy humans was evaluated. FM formulations were administered into a simulator of the human intestinal microbial ecosystem (SHIME^®^) to evaluate the viability of lactic acid bacteria (LAB), microbiota composition, presence of short-chain fatty acids (SCFA), and ammonium ions. The probiotic LAB viability in FM was affected by the addition of the fruit pulp. *Phocaeicola* was dominant in the FMPF and FMB samples; *Bifidobacterium* was related to FM formulations, while *Alistipes* was associated with FMPF and FMB, and *Lactobacillus* and *Lacticaseibacillus* were predominant in FMC. *Trabulsiella* was the central element in the FMC, while *Mediterraneibacter* was the central one in the FMPF and FMB networks. The FM formulations increased the acetic acid, and a remarkably high amount of propionic and butyric acids were detected in the FMB treatment. All FM formulations decreased the ammonium ions compared to the control; FMPF samples stood out for having lower amounts of ammonia. The probiotic FM with fruit pulp boosted the beneficial effects on the intestinal microbiota of healthy humans in addition to increasing SCFA in SHIME^®^ and decreasing ammonium ions, which could be related to the presence of bioactive compounds.

## 1. Introduction

The functional food properties are associated with the metabolic or physiological role that the nutrient or non-nutrient has on the growth, development, maintenance, and other normal functions of the human organism [1]. For the development of functional foods, milk and dairy products are the mainly used matrices, since they are consumed regularly, have high nutritional value, and exhibit several health benefits. They provide high quality proteins, bioactive peptides, and essential amino acids; are rich in calcium, which can prevent osteoporosis, and lipids rich in essential fatty acids, and are also sources of vitamins, among other nutrients [2].

Among functional dairy products, fermented products are relevant since the use of probiotic bacteria for carrying out fermentation is strategic when meeting the consumers’ demand for healthy yet appealing food products. Moreover, probiotic fermented milk (FM) can also modulate the gut microbiota, increasing the presence of beneficial microorganisms and an arsenal of essential metabolites [3,4].

Another trend in the development of functional dairy products is to supplement them with fruit pulp, which can improve sensory properties, such as taste, aroma, texture, and color, diversifying the products available on the market [5,6]. Tropical fruit pulp, especially from unconventional fruits—those that are not widely known or consumed by most of the population—contribute to the healthy appeal at the same time as increasing the nutritional value of the product given the bioactive compounds naturally found in them. Many of these bioactive compounds have antioxidant properties, which can contribute to increasing the shelf-life of the products and are related to a lower incidence of certain diseases [7,8] through the modulation of the intestinal microbiota.

Initially, studies focused on the effect of bio-compounds on pathogenic bacteria present in the microbiota; however, in recent years, research has focused on evaluating the effect of polyphenols (PP)—including fruits rich in bioactive compounds—on the modulation of the intestinal microbiota [9,10,11]. The balance that these bio-compounds provide when stimulating beneficial bacteria and decreasing pathogenic ones on the Firmicutes/Bacteroidetes ratio was the main object of study [12,13,14]. Probiotics are also known for having a modulating effect on the intestinal microbiota [15,16]; it is also known that dairy products, mainly probiotic FM, can function as coadjutants to improve the function of the gastrointestinal (GI) barrier and as immunomodulators to promote GI health [17,18]. However, research to evaluate the modulating effect of probiotic FM on the intestinal microbiota has gained prominence in recent years [4,17,19], although studies aimed at better understanding the modulation are still necessary, especially for FM supplemented with fruit pulp.

Therefore, the aim of this study was to evaluate the effect of fermented milk with and without fruit pulp on LAB viability, intestinal microbiota composition (richness, diversity, differential abundance, structure of the microbial communities), and production of short-chain fatty acids and ammonium ions by means of an in vitro test using the Simulator of Human Intestinal Microbial Ecosystem (SHIME^®^).

## 2. Materials and Methods

### 2.1. Lactic Acid Bacteria (LAB) Cultures

*Lacticaseibacillus casei* SJRP38 isolated from water buffalo mozzarella cheese and *Lactiplantibacillus plantarum* ST8Sh isolated from Bulgarian salami, both identified by 16S rRNA gene sequencing and characterized as potentially probiotic strains [20,21], were used. The strains were stored at −80 °C, in the presence of 20% sterile glycerol (*v*/*v*) as a cryoprotector. For revitalization, the isolated strains were inoculated (2%, *v*/*v*) in 10 mL MRS broth (Difco Laboratories, Detroit, MI, USA) at 37 °C for 18 h. Subsequently, the activated cultures were centrifuged (5000× *g*, 5 °C, 5 min) and washed twice with 0.85% saline solution before being inoculated in milk. The freeze-dried *Streptococcus thermophilus* TA 080 (Sacco, Cadorago, CO, Italy) was used as a starter culture directly in the product. These proportions yielded initial counts of approximately log 7 CFU/mL after milk inoculation.

### 2.2. Passion Fruit and Buriti Pulps Preparation

Passion fruits (*Passiflora edulis f. Flavicarpa Deg*.), originating from Bahia state, were purchased in São José do Rio Preto-SP, Brazil. The fruits were washed and sanitized in running water and submerged for 10 min in 10% (*w*/*v*) sodium hypochlorite (NaClO) solution (Synth, Diadema, SP, Brazil). Subsequently, the pulp was manually separated from the seed using a sieve. The pulp was then stored in polyethylene plastic bags in the absence of light and frozen (−80 °C). Buriti fruits (*Mauritia flexuosa*) were harvested and processed in Teresina-PI, Brazil, the typical region for buriti production. The fruits were washed and sanitized, as previously described, manually pulped using a stainless-steel knife, and stored at 4 °C, as described by Moura Filho [22]. Subsequently, the pulp was sent to São José do Rio Preto-SP, Brazil. Upon arrival, the pulp was frozen (−80 °C).

### 2.3. Preparation of the Cultures and Fermented Milk

To evaluate the effect of the fruit pulp on the gut microbiota, three different formulations were prepared: FM without pulp (control—FMC), FM with 1% (*w*/*v*) passion fruit pulp (FMPF), and FM with 1% (*w*/*v*) buriti pulp (FMB). The reconstituted milk (10% skim powder milk (Molico, Nestle, SP, Brazil) in sterile water) with or without fruit pulp, according to the experiment, was heated to 90 °C for 10 min using a food processor (Thermomixer TM 31, Cloyes-sur-le-Loir, France), cooled to inoculation temperature (37 °C), and distributed into sterilized bottles.

The activated cultures of *Lb. casei* SJRP38 and *Lb. plantarum* ST8Sh, prepared as described previously, were inoculated in the proportions of 2% and 0.1% for freeze-dried *Str. thermophilus* TA 080. For the fermentation process, the bottles were incubated in a water bath (EVLAB, Londrina, PR, Brazil) at 42 °C until reaching a pH of 4.6. After the fermentation was completed, the product was cooled and the curd was manually broken in a standardized way for all formulations; finally, it was cooled to 4 °C and then stored, as described by Borgonovi et al. [6].

### 2.4. Lactic Acid Bacteria Viability in Fermented Milk

For the analysis of LAB viability in FM, a 100 μL aliquot of all FM samples was withdrawn; serial dilutions up to 10^−8^ were prepared with 0.85% saline solution and plated to appropriate solid growth media on Petri dishes, according to Solieri et al. [23] with modifications: *Streptococcus* spp. were counted using M17 agar (Difco) under aerobic conditions at 50 °C, *Lb. casei* SJRP38 was counted in MRS agar (Difco) at 45 °C, and *Lb. plantarum* ST8Sh was counted in MRS agar (Difco) at 15 °C, both under anaerobic conditions, all after incubation for 48 h. The incubation temperature of *Lb. casei* and *Lb. plantarum* were defined after preliminary trials in which different incubation temperatures (15 °C, 25 °C, 30 °C, 37 °C, 45 °C, 50 °C, 55 °C) were assessed aiming to count both evaluated strains separately.

### 2.5. Simulator of Human Intestinal Microbial Ecosystem (SHIME^®^)

**Figure 1:**
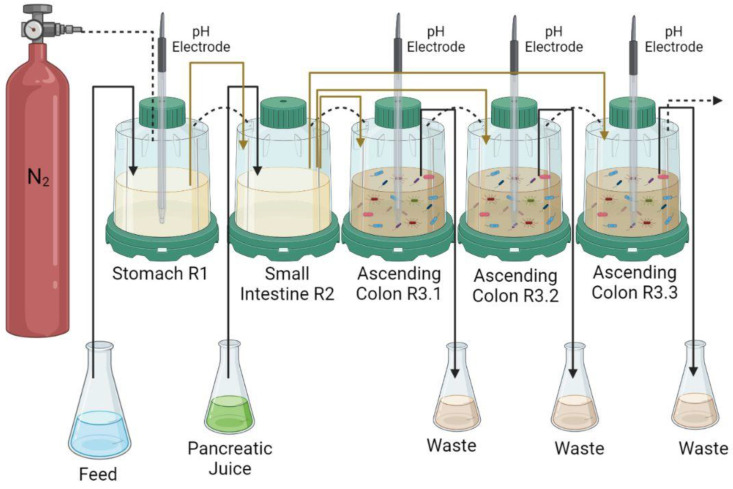
The Simulator of the Human Intestinal Microbial Ecosystem (SHIME^®^) is a computer-controlled reactor connected through peristaltic pumps and operated completely anaerobically by the addition of nitrogen. It consists of closed compartments representing the stomach (R1—pH 2.0–2.5 and pepsin digestion), small intestine (R2—pH 4.3–4.8, bile salts, artificial pancreatic juice, and absorption), and three reactors miming the ascending colon (R3.1, R3.2, and R3.3—pH 5.6–5.9).

The SHIME^®^ model was used to simulate the human digestion process. It is a computer-controlled system in which volumetric capacity, pH, temperature (37 °C), and retention time (24 h) were controlled. A schematic description of the SHIME^®^ system [24] is presented in Figure 1. This SHIME^®^ model consists of five compartments representing the stomach, small intestine, and ascending colon, anaerobically controlled by the addition of nitrogen and the pH value adjusted by using hydrochloric acid or sodium hydroxide, accordingly, in each reactor.

SHIME^®^ was adapted for this specific study, in such a way that the transverse and descending colon were replaced by the triplicate of the ascending colon, according to Salgaço et al. [25].

#### 2.5.1. Composition of the SHIME^®^ Feed Medium

The feed medium used in SHIME^®^ was prepared with distilled water, supplemented with 3 g/L starch (Maizena^®^, Unilever Brazil, SP, Brazil), 2 g/L pectin (Sigma-Aldrich, Burlington, MA, USA), 4 g/L gastric mucin type II swine (Sigma-Aldrich), 1 g/L xylan (Megazyme, Bray, Ireland), 1 g/L peptone (Kasvi, São José do Pinhais, PR, Brazil), 1 g/L arabinogalactan (Sigma-Aldrich), 0.4 g/L glucose (Synth, São Paulo, SP, Brazil), 3 g/L yeast extract (Kasvi), and 0.5 g/L L-cysteine (Sigma-Aldrich) [26]. 

Stomach conditions were simulated in reactor 1 (R1) and the pH value was adjusted by adding HCl, along with the carbohydrate-based medium. In order to reach the duodenum conditions, in the second reactor (R2), an artificial pancreatic juice was added, composed of 12.5 g/L sodium bicarbonate (LS Chemicals, Maharashtra, India), 6 g/L ox-bile (Sigma-Aldrich), and 0.9 g/L pancreatin (Sigma-Aldrich) [26]. This carbohydrate-based medium plays an important role in the environmental adaptation and inoculum growth with the formation of a stable and representative community [24].

#### 2.5.2. Microbiota Colonization

The SHIME^®^ reactors were colonized with a pool of feces donated by three healthy male volunteers, aged 18–22 years old. The following criteria were met: donors had not taken antibiotics within a period of six months prior to the study; had not consumed probiotic products over the past 3 to 6 months; and did not have any food allergies or intolerance to dairy products. The stool inoculum was prepared according to the procedures described by Duque et al. [27]. The samples (40 g) were collected on 3 different days, homogenized, diluted in 200 mL phosphate buffer (pH 6.5), which was composed of 7.08 g/L monosodium phosphate (Synth), 5.98 g/L disodium phosphate (Synth), and 1 g/L sodium thioglycolate (Merck, Darmstadt, HE, Germany). This mixture was homogenized in the stomacher and centrifuged at 3000× *g* for 15 min. The supernatant was collected, and 10 mL was added to the three compartments that simulated the ascending colon (R3.1, R3.2, and R3.3, Figure 1) along with 500 mL of sterile feed medium. For stabilization of the SHIME^®^ system, the feed medium (240 mL) and the pancreatic juice (60 mL) were placed into the three compartments for 14 days before the experiments, according to Possemiers et al. [26] and Van de Wiele et al. [28].

#### 2.5.3. Experimental Protocol

After 14 days of stabilization of the human microbiota, the FM formulations (FMC, FMPF or FMBP) were administered for 5 days, followed by 5 days washout, as described by Duque et al. [27]. The treatments consisted of 80 g FM and 240 mL feed medium. During the washout period, only the feed medium (240 mL) and the pancreatic juice (60 mL) were added. After the treatments and washout of FMC, the other formulations (FMPF and FMB) were added separately under the same conditions. The volumetric capacity, pH, temperature (37 °C), and retention time (24 h) were controlled in the last three compartments [26] and stirred with a magnetic stirrer throughout the entire process. The anaerobiosis of the system was achieved with the addition of nitrogen and the pH corrected in each vessel with hydrochloric acid or sodium hydroxide, reaching the pH range of 5.6–5.9 [26]. Samples were collected in triplicate from the ascending colon (last 3 reactors) on two different days. During the stabilization period, samples were collected on the 13th and 14th days, whereas during treatment administration (FMC, FMPF and FMB) and washout periods, samples were collected on the 4th and 5th days.

### 2.6. Evaluation of Metabolites Production in SHIME^®^

The short-chain fatty acid (SCFA) levels and ammonium ions were determined from samples collected from reactors representing the ascending colon and frozen at −20 °C. This analysis was performed according to Espírito Santo et al. [29] and Adorno et al. [30]. Briefly, the fatty acids were extracted with diethyl ether and analyzed in a gas chromatograph equipped with a flame ionization detector. For ammonium ions (${\mathrm{NH}}_{+}^{4}$), after the digestion process in the SHIME^®^ simulator, the samples were collected and quantified using a specific ion meter (Model 710A, Orion, Thermo Fisher Scientific, Waltham, MA, USA) coupled to a selective ammonia ion electrode (Model 95–12, Orion), according to Bianchi et al. [31].

### 2.7. Microbial 16S rRNA Gene-Based Sequencing and Bioinformatic Analysis

The determination of the composition of the intestinal microbiota collected from the SHIME^®^ was performed using next-generation sequencing by Neoprospecta Microbiome Technologies (Florianópolis, SC, Brazil). The V3–V4 hypervariable region of the 16S rRNA gene was amplified using specific primers. The data were organized by microbiota phyla, families, genera, and species, according to Casarotti et al. [4].

Initially, the DNA was extracted from 2 mL of samples collected (as described in item 2.4.3) from the reactors that represent the ascending colon in duplicate (total of 42 samples), using the QIAamp DNA Stool Mini Kit (Qiagen, Hilden, Germany), according to the manufacturer’s protocol, and visualized by agarose gel electrophoresis 1% stained with SYBR^®^ Safe (Invitrogen, Carlsbad, CA, USA). Next, the DNA quantification was conducted (Qubit^®^ 3.0 Fluorometer–Life Technologies, Carlsbad, CA, USA) based on the ratio 260/280; the quality of the DNA was verified by using the Nanodrop ND-1000 spectrophotometer (Nanodrop Technologies, Wilmington, DE, USA). The libraries were sequenced using the equipment MiSeq Sequencing System (Illumina Inc., San Diego, CA, USA).

After sequencing, the FastQC software was used for the initial assessment of the quality of the sequenced data. Additionally, the libraries were analyzed [32] to detect the presence of residual bases and amplification primers, which were then removed [33]. The final portion of the sequences was removed to ensure better quality [34]. Bases with a window consisting of 3 consecutive bases with a Phred (Q) quality value <20 and complete readings with a total mean <Q20 were removed.

Superior quality reads were submitted to the DADA2 pipeline [35] using the R software (v.4.1.2; R Core Team). Initially, the reads were filtered and truncated, considering an expected error of 2 and a minimum size of 283 bp. Then, the error probabilities in bases were estimated, and the sequences were corrected based on the model obtained. Thus, amplicon sequencing variants (ASVs) present in each sample were designated, which were then investigated and filtered for the presence of possible chimeric sequences. The ASVs were annotated against the RDP reference sequence database v. 18 [36] and the taxonomies were assigned to each ASV. The ASVs prevalent in only a single sample were disregarded.

The rarefaction curves were obtained to evaluate the effectiveness of the sampling depth [37]. Relative abundance was analyzed for phyla, families, and genera. Alpha diversity was estimated by investigating the richness and diversity measures (Shannon and Gini-Simpson index) [38].

### 2.8. Statistical Analysis

The bacteria viability analyses in the FM, as well as ammonium, SCFA, and viability of bacteria in the reactors representing the ascending colon in SHIME^®^, were all carried out by analysis of variance (ANOVA) followed by a Tukey test, which was used to compare the formulations. A significance level of 5% probability and Minitab 16 software were adopted.

The Kruskal–Wallis test (*p* ≤ 0.05) was used to compare differences in richness and α-diversity among FM formulations. In case of significance, the Fisher’s LSD post-hoc test was used for pairwise comparison and grouping of means (*p*≤ 0.05). Both analyses were conducted using the R software [39].

The beta diversity analysis was performed by calculating Bray–Curtis dissimilarities between samples taken from the FM formulations using the R software. For each cluster, a PERMANOVA analysis [40] was performed to assess whether there was a difference among formulations (*p* ≤ 0.05). In the event of a difference, a post-hoc analysis was performed [41] to assess which formulations differed from each other. To reduce the multidimensionality of the distances, a principal coordinate analysis (PCoA) was performed.

DESeq2 was used for the evaluation of differentially abundant taxa [42] by comparing the means based on a negative-binomial model using the Wald test (adjusted *p*≤ 0.05). The analyses were graphically represented with the R software [43]. For this analysis, two sets of comparison were considered: (1) only FM formulations and (2) serialization of formulations according to the previous control (FM) washout.

The structural characteristics of microbial communities from the samples of FM formulations were evaluated based on co-occurrence networks considering the taxonomic level of the genus. Those whose relative abundance was lower than 0.1% were filtered out. The nets were obtained independently for each treatment (only fermented milk preparations were considered for this analysis). After calculating Pearson’s coefficients [44], a minimum threshold of Pearson’s coefficient of ±0.75 was considered among the significant correlations (*p* ≤ 0.05) in order to restrict the scope of networks only to strongly positive or negative relationships. The construction of the networks and the topological information were obtained through the R software v.1.3.4 [45].

Correlations between the microbiota and the levels of SCFAs and ammonia were obtained considering the taxonomic level of family. Pearson’s coefficients were calculated using the R software [44], and for significant correlations (*p* ≤ 0.1), linear regression graphs were obtained.

## 3. Results and Discussion

### 3.1. LAB Viability in Fermented Milk

The fruit pulp did not interfere in LAB viability in FM. The population of *Lb. casei* in the FM with pulp fruits was lower than that in FMC (Table 1). A similar result was previously observed by Borgonovi et al. [6], in which a synergistic relationship between fruit pulp and the LAB in FM with pulp fruit was not observed. This type of interaction between LAB and fruit may vary according to the strain and/or type of fruit. However, Espírito-Santo et al. [29] showed a positive effect resulting from mixing probiotic strains (*Lactobacillus acidophilus* L10, *Bifidobacterium animalis* subsp. *lactis* Bl04, and *Bifidobacterium longum* Bl05) and açaí pulp. The beneficial effect of fruit pulps on the viability of LAB was ascribed to the presence of bioactive compounds, such as phenolic compounds, carotenoids, and organic acids, in addition to fibers, such as fructooligosaccharides (FOS) found in fruits, some of them described as prebiotics [5,46].

The viability of *Lb. plantarum* ST8Sh in FM was similar between the type of fruit and the difference was small compared to the control (Table 1). Moreover, the viability of *Str. thermophilus* TA080 was not affected by the addition of fruit pulp to the FM. These differences in the growth of *Lb. plantarum* ST8Sh and *Str. thermophilus* TA080 in FM can be explained by the compounds present in the milk base, which may stimulate or inhibit the viability of these strains. Additionally, the total solids present in the FM can alter the viability of the LAB strains.

Despite the lower viability of LAB in FMPF and FMB, the total probiotic count in all FM formulations meets the minimum required value of ≥6 log CFU/mL for consumption [5]. The fruit pulp present in probiotic fermented foods is considered to be a beneficial ingredient, since it may protect the probiotics from adverse conditions found in the human GI tract environment. It can also have a potential prebiotic effect, a relevant factor in bacterial growth and proliferation, acting in synergy with probiotics in the human intestines after their ingestion [47].

### 3.2. Microbiota Composition Evaluation by Gene Sequencing

#### 3.2.1. Data Processing

A total of 4,626,432 reads were generated after the sequencing of 42 samples. After quality control, processing, and filtering of ASVs, the reads were sufficient to cover the diversity of the studied conditions since there was a stabilization in the rarefaction curves (Supplementary Figure S1). In total, 481 ASVs were identified. Regarding classifying capacity, 92.3% of the usable sequences could be classified up to the genus level, and we found 4 phyla, 11 classes, 19 orders, 33 families, and 70 genera.

#### 3.2.2. Taxonomic Profile and Ecology

**Figure 2:**
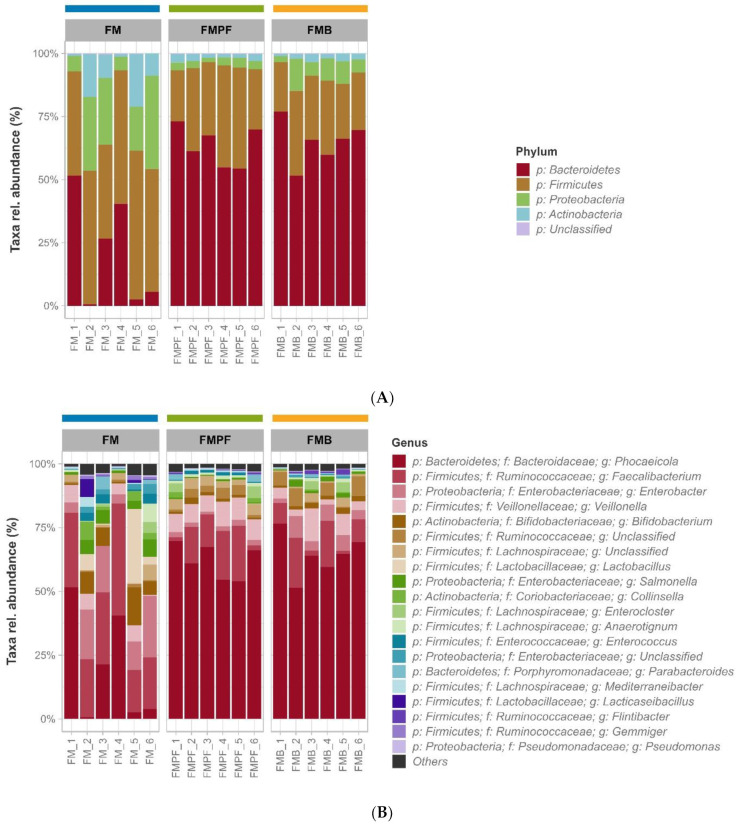
Taxonomic profiles of communities associated with fermented milk without the addition of fruit pulp (FM_1 to FM_6) and with the addition of passion fruit pulp (FMPF_1 to FMPF_6) or buriti pulp (FMB_1 to FMB_6). Relative abundance of phyla (**A**) and 20 most frequent bacterial genera (**B**). Less abundant genera were grouped under category “Others”.

The phylum Bacteroidetes showed greater abundance in the microbial communities associated with the FM formulations, with mean values of 25.13%, 63.15%, and 65.31% in FMC, FMPF, and FMB samples, respectively (Figure 2A). Firmicutes were also highly abundant; this phylum was predominant in FMC samples (average of 49.26%) and the second most prevalent in FMPF and FMB samples (averages of 31.51% and 25.30%, respectively). The phyla Proteobacteria and Actinobacteria were especially abundant in the FMC samples, with averages of 17.37% and 8%, respectively. For FMPF samples, the average of these genera represents less than 5% of the microbiota, while for FMB, this value is slightly higher than 9%.

The Firmicutes phylum includes many genera, such as Bacillus, Streptococcus, Leuconostoc, Listeria, Roseburia, Lactobacillus, Veilonella, Clostridium, Oscillospira, Lachnospira, Leuconostoc, and Enterococcus, among others. This phylum includes bacteria with Gram-positive cell wall structure and low G+C in the DNA [3,4]. The Bacteroidetes phylum is the second most populous in the human gut, with the predominance of the Bacteroides and Prevotella genera. Bacteria species form metabolites or low molecular weight compounds, such as SCFAs produced from the fermentation of dietary fibers. The decreased ratio of Firmicutes and Bacteroidetes in microbiota is associated with a healthier composition [12,13].

FMPF and FMB formulations increased Firmicutes and Bacteroidetes, and all formulations increased the Actinobacteria and decreased the Proteobacteria phyla compared to their respective washout-control treatments (Supplementary Figure S2). The increase in the Firmicutes phylum observed for FMPF and FMB compared to their washout counterparts may be due to the presence of fibers in the fruit pulp [48].

The increase in the Actinobacteria phylum is another positive outcome observed during the administration of the FM. This phylum is related to a healthy microbiota, since it harbors the main bacteria that play a protective role in maintaining intestinal barrier homeostasis [49], such as acetate producing *Bifidobacteria* [50]. On the other hand, the decrease in the Proteobacteria phylum is desirable, since this phylum, which includes the genera *Clostridium* and *Bacillus,* is related to the incidence of inflammatory diseases [51] and is known for its proteolytic activity [52]. In the present study, the decrease in Proteobacteria may be related to the lower availability of amino acids, which may have been metabolized by LAB with proteolytic activity during the fermentation process of the FM. Oddi et al. [53] also reported modulation of the intestinal microbiota with the reduction of Proteobacteria, mainly those from the family *Enterobacteriaceae* and from the genera *Escherichia, Shigella*, and *Clostridium*_sensu_stricto_1, when probiotic strains of *Lb. plantarum* 73a alone or in combination with *B. animalis* subsp. *lactis* INL1 were administered in SHIME^®^.

Considering that the increase in the Firmicutes and Actinobacteria phyla and decrease in the Proteobacteria phylum may be related to eubiotic microbiota, we suppose that this microbiota remained in homeostasis during the administration of the FM. In addition, the initial microbiota (control) was composed of Firmicutes (average of 55.37%), Proteobacteria (average of 28.06%), and a minimal proportion of Actinobacteria (average of 2.15%), when it is desirable that Firmicutes and Bacteroidetes compose 85–90% of the total microbiota and, less abundantly, Actinobacteria, Proteobacteria, and Verrucomicrobia [54], differently from the microbiota observed during and after the administration of FMPF and FMBP, in particular.

Regarding the taxonomic profiles, there was a similarity between the samples of FM supplemented with fruit pulp (FMPF and FMB) when compared to samples of FM without any fruit pulp (FMC), both at the phylum (Figure 2A) and the genus levels (Figure 2B). It is also noteworthy that the FMC treatment showed a more diffuse relative abundance among the most prevalent genera, while the FMPF and FMB samples showed more homogeneous profiles and were dominated by the most prevalent genera (Figure 2B).

It was observed that *Phocaeicola* presented the highest general abundance, with averages of 24.49%, 61.91%, and 64.61% in FMC, FMPF, and FMB samples, respectively (Figure 2B); however, the species identity distribution in the samples was not identified. Although a large number of bacteria, archaea, viruses, and unicellular eukaryotes reside in the human GI tract, only some bacterial genera dominate the human gut, including *Bacteroides, Clostridium, Bifidobacterium,* and *Faecalibacterium* [55,56] The *Phocaeicola* genus comprises Gram-negative, strictly anaerobic bacteria, generally associated with human fecal samples [57,58]. *Bacteroides* and *Phocaeicola* represent 30% of the commensal intestinal microbiota, playing an essential role in the gut ecosystem [59,60]. Moreover, *Phocaeicola* species present glycosyl hydrolases enzymatic activities, which degrade mucin glycans and many plant-derived heteropolysaccharides [61,62,63], generating important metabolites such as succinate and propionate [64,65]. There is robust evidence that these gut-associated microbes are involved in the prebiotic metabolisms and bioactive compounds synthesis, which are frequently associated with human and animal health benefits [59,66]. More precisely, from the aforementioned *Phocaeicola*, a bacterial species of special interest is *P. vulgatus*, the most abundant microbe within the *Bacteroidaceae* family normally found in the colon, constituting up to 10^10^ cells per gram of stool [59].

In the FMC samples, the *Faecalibacterium* genus was more abundant (average of 28.63%), although this was relatively heterogeneous among the samples of this treatment (Figure 2B). The *Faecalibacterium* genus encompasses the *Clostridium leptum* cluster and represents the second most dominant group in the human gut [67,68]. *Faecalibacterium prausnitzii* represents approximately 5% of the total fecal microbiota in healthy adults and produces metabolites from glucose/prebiotic fermentation, such as formate, D-lactate, acetate, and high levels of butyrate [55,69,70]. In addition, *F. prausnitzii* is well adapted to the gut ecosystem, where it is possibly cross-fed by other microorganisms from the gut microbiota [69,71].

**Figure 3:**
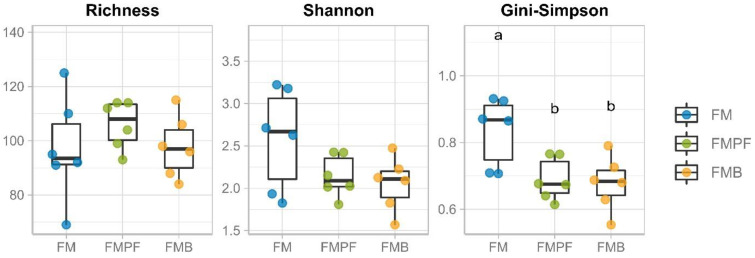
Richness and alpha-diversity (Shannon and Gini–Simpson). Comparisons of milk averages were made. Distinct letters indicate significant differences between conditions.

The values of richness and alpha diversity are shown in Table 2. The comparison between the alpha diversity measures is in accordance with that observed in the taxonomic profiles (Figure 2), since the FMPF and FMB formulations had lower bacterial diversity (Gini–Simpson) in relation to the FMC treatment (Figure 3), reflecting the clear dominance observed in the samples under these conditions. However, the species richness and Shannon diversity obtained for these formulations were not significantly affected by the addition of the fruit pulp.

There are scarce studies evaluating the impact of fermented milk with fruit pulp on alpha diversity indices (richness and evenness) for in vitro simulation models. However, we expected that the alpha diversity indices would increase in FMPF and FMB, since the increases in these indices are related to intestinal health [72]. A previous study, using an in vitro simulator of the GI tract (TIM-2 system), showed increased microbiota alpha diversity when treated with orange bagasse and passion fruit peels [73]. 

**Figure 4:**
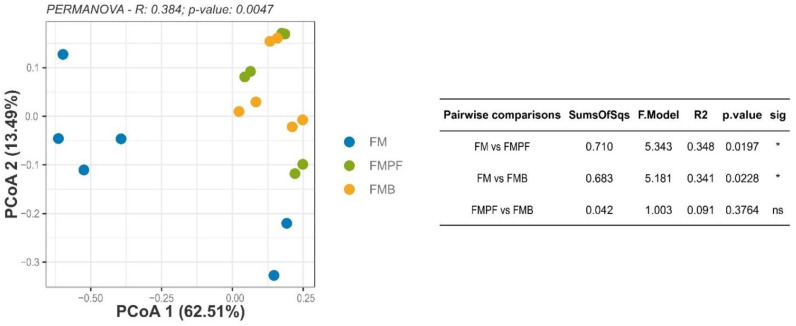
Principal coordinate analysis (PCoA) of Bray–Curtis dissimilarities distances. The aggregated table (right) shows which sample groups differed according to the significance observed by the PERMANOVA post-hoc test. * *p* ≤ 0.05.

The beta-diversity analysis evaluated the presence of differences among the microbiota composition resulting from FM formulations (Figure 4). According to the PERMANOVA test, a difference was observed in the composition of the microbiota among the various FM formulations. The post-hoc test revealed a significant difference between the FMC and FMPF formulations (*p* = 0.0197), as well as between the FMC and FMB (*p* = 0.0228). Among the formulations with fruit pulp, there are no significant differences (*p* > 0.05). Furthermore, the reduction in dimensionality obtained by PCoA resulted in an explanation of 76% of the variability found in the samples.

Since there were differences in the beta diversity between FM and those with added fruit pulp, we may suggest that the FMPF and FMB were able to modulate the microbiota in vitro. Our research group already showed in vitro microbiota modulation by fermented goat milk with passion fruit [4].

Fruits are a source of carbohydrates, fibers, amino acids, acids, minerals, polyphenols, vitamin C, B-complex vitamins, provitamin A, carotenoids, phytosterols, aromatic compounds, and other bioactive compounds that are beneficial to human health [74,75,76]. The impact of these bioactive compounds on the intestinal microbiota and the metabolites generated need to be considered to better understand the role of these components and the addition of fruit pulp in microbiota modulation [77,78,79]. A balanced microbiota (eubiosis) confers benefits to the human health, and alterations in microbiota diversity and function (dysbiosis) are associated with the development of some diseases, including metabolic, cardiovascular, chronic inflammatory, and neurodegenerative diseases [80,81,82,83].

#### 3.2.3. Differential Abundance

**Figure 5:**
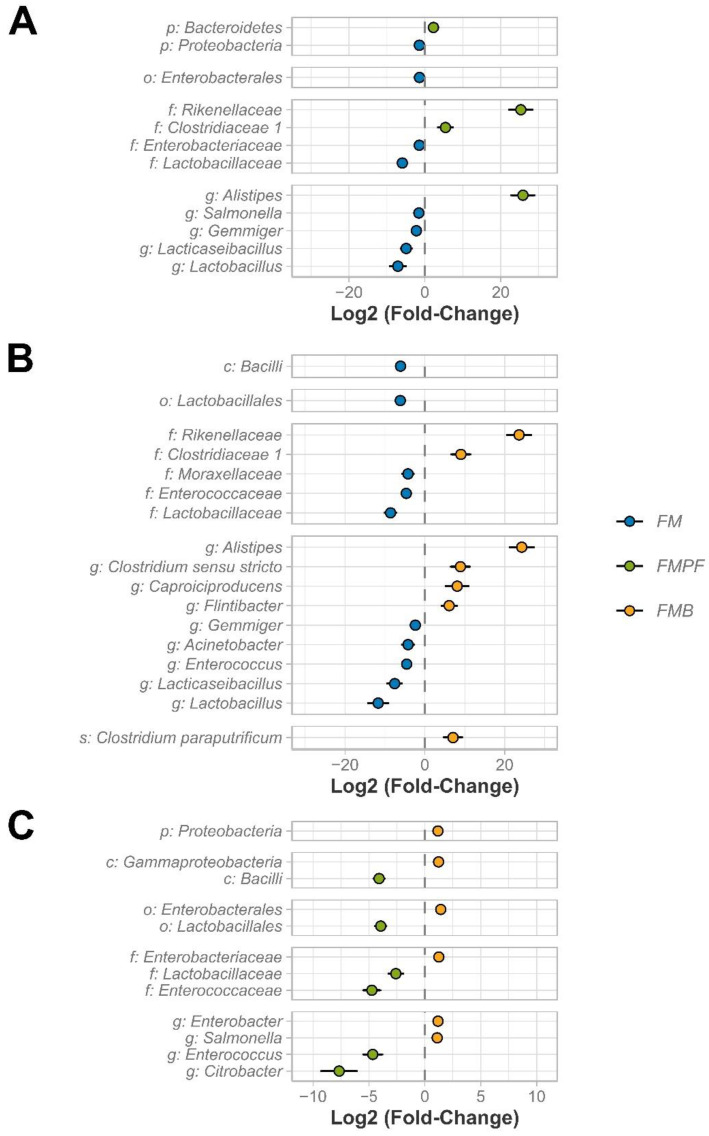
Differential abundant (DA) taxa among fermented milk treatments. The dot plots represent the mean intensity of the difference (fold-change) complemented by the standard error. The taxa are preceded by letters that represent the taxonomic level, namely, phylum (p), class (c), order (o), family (f), genus (g) and species (s). Comparison between FMC vs. FMPF (**A**), FMC vs. FMB (**B**) and FMPF vs. FMB (**C**).

To detect differences between taxa, two different comparisons were used: (1) comparison between fermented milk formulations (FMC vs. FMPF, FMC vs. FMB, and FMPF vs. FMB—Figure 5), and (2) each fermented milk against its respective control (CTRL vs. FMC, WFM vs. FMPF, and washout of FMPF–WFMPF) vs. FMB—Supplementary Figure S3). In comparison 1, 41 differentially abundant taxa (DA) were identified. In comparison 2 (FM vs. respective control), 102 DA taxa were identified, respectively, 36, 36, and 30 for samples related to FMC, FMPF, and FMB formulations. In the comparison between FM formulations, the most significant differences noted were regarding the *Clostridiaceae* (Group 1) and *Rikenellaceae* families, in addition to the *Alistipes* genus, which were higher in milk with fruit pulp (FMPF and FMB). On the other hand, the *Lactobacillaceae* family, as well as its associated genera *Lacticaseibacillus* and *Lactobacillus*, were depleted in these same formulations when compared to FMC (Figure 5). Members of LAB were expected to be greater in fermented milk alone. We can suggest that the addition of fruit pulp may modulate the microbiota in vitro and contribute to the expansion of other gut ecosystem microbes, such as the *Clostridiaceae* and *Rikenellaceae* families, in addition to the *Alistipes* genus.

The *Clostridiaceae* family comprises spore-forming microorganisms with approximately 100 species. The *Clostridium* species are found ubiquitously in the environment, soil, water, and in the human gut as part of the commensal microbiota [84]. Special characteristics from the *Clostridium* species include co-fermentation of pentose and hexose sugars, high ethanol yield, and cellulolytic activity [85]. In our analyses, we detected unclassified *Clostridium* species but also *Clostridium sensu stricto*, obligate anaerobes that dissimilate glucose into acetate, butyrate, lactate, ethanol, H_2_, and CO_2_. In a study by Kong et al. [86], decreased microbiota diversity and beneficial butyric acid-producing microbes were reported in obese mice treated with a high-calorie diet, including *Clostridium sensu stricto*, suggesting their importance in providing energy for colonocytes and protection of the epithelial barrier.

The *Rikenellaceae* family presents Gram-negative rods, non-motile, non-spore forming bacteria with anaerobic metabolism, and bile resistance. The *Alistipes* species are part of this family found in the GI tract of a number of different animals [87,88]. This bacterium can hydrolyze tryptophan to indole and the main fatty acid produced is 13-metyltetradecanoic acid [89].

The *Lactobacillaceae* family, generically called “lactobacilli” until 2020, includes rod-shaped or coccobacilli Gram-positive, non-motile, non-spore forming, facultatively anaerobic bacteria. Based on their ability to hydrolyze carbohydrates, they are classified into homofermentative or heterofermentative species, which convert carbohydrates into lactic and acetic acids, ethanol, and CO_2_ [90,91]. The members of this family are found in oral, GI, and urogenital commensal microbiota, as well as in water, soil, and food, including dairy products, fruit, grain, meat and fish products, beer, wine, and pickled vegetables [90].

Although in this study, the *Lactobacillus* species were not identified, the *Lactobacillus* genus comprises 25 phylogenetic groups, at the phenotypic, ecological, and genotypic levels [92]. Similarly, even though *Lacticaseibacillus* was unclassified in our samples, it represents one of the largest genera among the LAB from the *Lactobacillaceae* family [92,93]. The impacts of fermented dairy products on human health can be wide-ranging depending on the species and fermentation processes involved. LAB, especially *Lactobacillus*, have been used in milk fermentation due to their ability to convert carbohydrates into organic acids and improve product quality. The benefits of fermented foods for human health involve the food–gut axis through interactions of ingested live microorganisms with the host, the probiotic effect, or indirectly, as a result of the ingestion of microbial metabolites synthesized during fermentation as a biogenic effect [91,94].

In the comparison between each FM and its control (Supplementary Figure S3), the consistent enrichment of the *Bifidobacterium* genus and its related higher taxonomic levels (Family: Bifidobacteriaceae; Order: Bifidobacteriales; and Class: Actinobacteria) stands out. In addition to being enriched in the three types of FM, this taxon is among the most prevalent genera in these samples (Figure 2B), which can be related to the ability of the *Bifidobacteria* to metabolize carbohydrates, such as the galactooligosaccharides (GOS) produced from lactose through the transgalactosylation activity of the β-galactosidase enzyme. GOS degradation occurs more effectively if associated with probiotic cultures, forming a synergistic combination with some species of *Bifidobacteria* and *Lactobacillus* [95].

#### 3.2.4. Structure of the Microbial Communities

**Figure 6:**
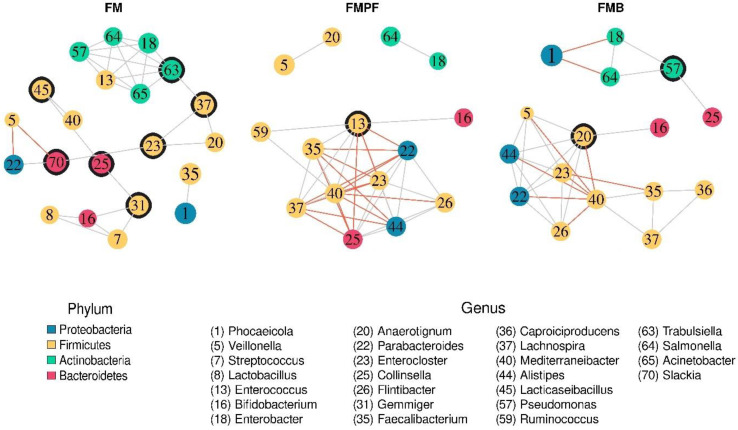
Co-occurrence networks of the genera identified by type of fermented milk treatment. Pearson’s correlation coefficients (r = ±0.75) were used at 95% confidence (*p* < 0.05). Positive and negative relationships are represented by links in gray and red, respectively. The filling color of the nodes represents the phylum to which the genus belongs. Articulation points (nodes whose absence would break the continuity of the modules) are highlighted with an external line.

Finally, the structuring of the microbiomes of the different types of fermented milk was evaluated by obtaining co-occurrence networks. It was observed that the type of fermented milk exerted changes in the relationships between the identified bacterial genera (Figure 6; Table 2). It is possible to identify a certain similarity between the networks of the FMPF and FMB formulations, while the FMC treatment presents a different configuration from the others. In this sense, the presence of larger modules with an incidence of negative relationships in the FMPF and FMB formulations is noted, while the FMC treatment has fragile modules with a high prevalence of seven points of articulation and a lower average of connections (average: 3048) compared to the other formulations. This characteristic is reinforced by a greater tendency towards modularity and average interconnectivity (“Mean betweenness”; Table 2) in the FMC treatment.

Furthermore, there is a change in the genera considered central in the networks (Figure 6; Table 3). The genus *Trabulsiella* (ID 63) was the central element of the FMC without significant co-occurrences in the FMPF and FMB formulations. The networks of FMPF and FMBP formulations show the same central genus: *Mediterraneibacter* (ID 40). In addition, the genus *Mediterraneibacter* has a series of conserved and consistent relationships in both networks arising from FM supplemented with fruit pulps, namely: positive relationships with the *Lachnospira* (ID 37) and *Faecalibacterium* (ID 35) genera and negative relationships with the *Alistipes* (ID 44), *Parabacteroides* (ID 22), *Flintibacter* (ID 26), and *Enterocloster* (ID 23) genera. Another notable feature is the positive association between the *Salmonella* (ID 64) and *Enterobacter* (ID 18) genera, which was consistent across the three networks.

The knowledge on the metabolic interspecies interactions and their functional roles is crucial for improving our understanding and predicting functioning and stability of the human intestinal microbiome in health and disease. However, we are still just beginning to understand how those bacterial species interact; knowing which potential interventions can help make us healthier remains a challenge. These interactions are highly dynamic and can result in competition for resources and/or cross-feeding for cooperative action [96,97].

The bacterial species representing the genus *Trabulsiella*, which were detected in FMC, are Gram-negative microorganisms from the Enterobacteriaceae family and Proteobacteria phylum [98]. Similar to the effect of FMC, probiotics and synbiotics supplementation increased the abundance of *Trabulsiella* and other genera, showing their role in maintaining gut health by lowering luminal pH and digestion of complex polysaccharides in chickens [98]. In addition, *Trabulsiella* representatives were detected in skin microbiome structure between healthy individuals from different races and ethnicity, especially on arms and hands [99]. It was also detected in unfermented and fermented rice washing water [100].

FMPF and FMBP formulations, nevertheless, showed a different microbial interaction. In a previous study, it was shown that buriti and passion fruit pulps are rich in bioactive compounds, such as phenolic compounds and carotenoids, in addition to fibers. The buriti pulp contains higher amounts of flavonoids compared to the passion fruit pulp; it is also rich in quercetin, while the major compounds of the passion fruit pulp are orientin, followed by vitexin. The fruit pulps are rich in carotenoids, and β-carotene is the major carotenoid in both pulps [6]. These compounds are known to modulate positively the intestinal microbiota.

*Mediterranneibacter* was the central genus in FMPF and FMB formulations. Representatives of this genus are Gram-positive, coccoid, or coccobacilli, non-motile obligate anaerobes belonging to the Lachnospiraceae family from the Firmicutes phylum. All type species were discovered in the human gut and the major metabolic products from carbohydrates were acetic, formic, and lactic acids [101]. In a cooperative action, *Mediterranneibacter*, *Faecalibacterium prausnitzii*, and *Lachnospira* sp. preferentially use these organic acids as a carbon source for butyrate production. An increase in the abundance of the *Mediterranneibacter* genus as characterized by the FMPF and FMB formulations was also reported by Ruiz-Rico et al. [102] when the impact of different modes of presentation of chitosan were assessed. Additionally, the abundance of *Mediterranneibacter faecis* is considered a biomarker for improving health due to its capacity to produce several short-chain fatty acids.

*F. prausnitzii* has specialized enzymatic machinery to secrete enzymes that break down the fiber polymers into fructose, glucose, and other monosaccharides, which are available as a carbon and energy source for the human colonic epithelium or other bacteria in the community, thereby cross-feeding them and improving gut health. Moreover, *F. prausnitzii* can produce large amounts of SCFAs, especially butyrate, which is secreted into the intestinal lumen [103]. Bacteria from the *Lachnospiraceae* family were observed to maintain the integrity of the intestinal barrier in mice, and their abundance was negatively correlated with chronic kidney disease [104].

Regarding the negative relationship of *Mediterraneibacter*, we hypothesize that the bacteria from this genus may competitively interact with *Alistipes, Flintibacter, Parabacteroides,* and *Enterocloster. Mediterraneibacter* and *Flintibacter* use the same substrates to produce SCFA, such as butyrate, while *Parabacteroides* utilizes polysaccharides and produces SCFAs via the metabolic pathway of fatty acid biosynthesis [105]. These interactions contribute to the modulation of the gut microbiota and improve the metabolic outcomes.

*Alistipes* is a member of the Bacteroidetes phylum, Rickenellaceae family, known to be an SCFA producer; its low abundance is associated to patients with non-alcoholic fat liver disease and liver fibrosis. However, it may be pathogenic in anxiety disorders, chronic fatigue syndrome, and depression, although it exerts a protective role in the health phenotype [104]. The *Flintibacter* genus is characterized by rod-shaped, Gram-negative bacteria from the Ruminococcaceae family. The type species for this genus is *Flintibacter butyricus.* It is able to produce butyrate and acetate not only from sugars but also from the amino acids glutamine and glutamate and is considered part of the beneficial microbiota [106,107]. *Mediterraneibacter glycyrrhizinilyticus* and *Enterocloster* were linked to secondary bile acid metabolism. Bile acids are potent antimicrobials and play a key role in the innate immune defense within the intestines [108].

A positive interaction between bacteria from the *Salmonella* and *Enterobacter* genera was observed in the SHIME^®^ system. Considering that bacteria from the Enterobacteriaceae family are the major groups present in the gut and have similar metabolic traits, it is possible that these bacteria use the same antibacterial antagonistic mechanisms, such as colicins, microcins, T6SS specialized protein secretion systems, and contact-dependent growth inhibition to counteract *Salmonella* in the gut. These bacterial interactions take place in a highly complex chemical environment, composed of chemical substances [109,110]. Additionally, the characterization of the interactions between the gut microbiota and host plasma metabolites could provide crucial insights into the effects of the gut microbiota on human health.

### 3.3. Short-Chain Fatty Acids and Ammonium Ions

There was a significant increase in acetic acid during the treatment periods in all types of FM and a decrease in its levels during washout (Table 3). A significant and remarkably high amount of propionate was produced with the addition of FMB. The highest amount of butyric acid was produced when FMC or FMB was added into SHIME^®^ (Table 3). Feng et al. [111] reported that the main genera of acetate-producing bacteria are *Bifidobacterium* spp., *Prevotella* spp., *Akkermansia* ssp., *Blautia hidrogenotrophica*, *Lactobacillus* spp., and *Bacteroides* spp. This explains the high production of acetate in all FM formulations, as there was an increase in *Bifidobacterium,* which produce acetate in these formulations, compared to their controls (Supplementary Figure S3). The increase in SCFA as a result of fermented milk administration was previously reported [112,113].

Regarding ammonia, FMPF and FMBP formulations had lower levels compared to FMC; FMPF samples had the lowest amounts of ammonia (Table 4). The lower ammonia production during the administration of FM containing fruit pulps observed in the present study is considered beneficial, since when present in high concentrations, in addition to being toxic to the organism and altering the cellular morphology of colonocytes, ammonia is related to carcinogenesis in the intestinal tissue, which increases the probability of cancer development [114,115]. When in the bloodstream, it can be linked to hepatic encephalopathy, as well as to neurotoxic effects [116]. The production of ammonia ions is related to the hydrolysis of urea and deamination of amino acids by the bacteria present in the intestines, correlating the increased production of ammonia with diets rich in protein [117,118].

Factors such as the type and amount of protein found in the matrix [52], as well as the use of probiotic strains in the fermentation of fermented products, may have had an important effect on preventing ammonia production. Moreover, the presence of phenolic compounds in fruit pulps can also contribute to the reduction of colonic protein fermentation [119].

Equivalent results for metabolites production after administering fermented milk were reported by Freire et al. [120]. These authors observed that fermented goat milk, with or without grape pomace extract, had a positive effect on the metabolism of the intestinal microbiota, increasing the production of SCFA and decreasing the concentration of ammonium. Rodrigues et al. [116] reported an increase in SCFA in SHIME^®^ by using healthy human microbiota after administering ice cream containing *Lb. acidophilus* and *B. animalis,* in addition to a decrease in ammonia after administering a dietary supplement containing *Lb. acidophilus* and *B. animalis* (both after 7 days of treatment).

**Figure 7:**
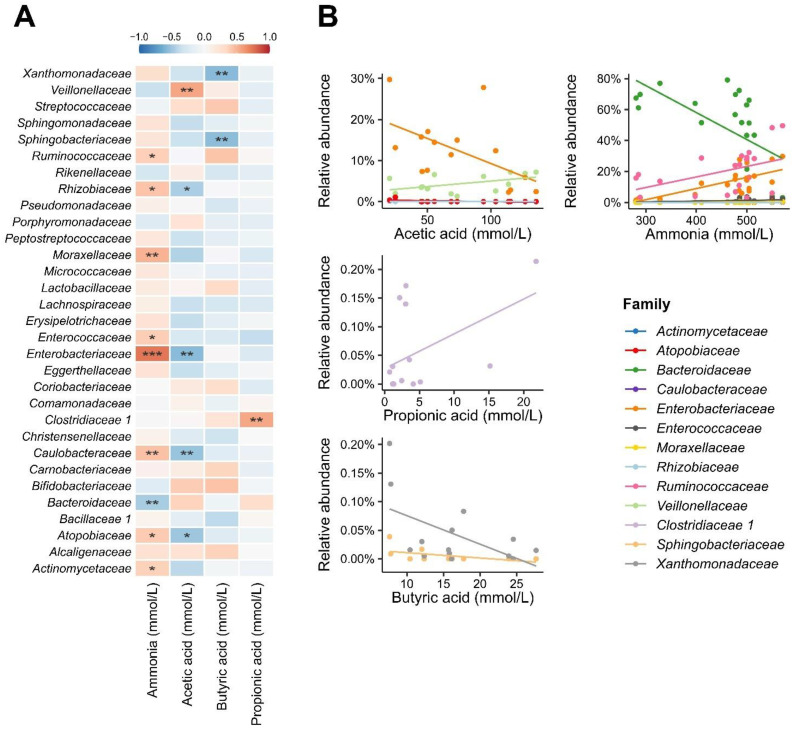
Correlation analysis between bacterial families and levels of ammonia and fatty acids. The heatmap (**A**) illustrates the values of the correlations in intensity and direction (positive or negative). The significance of the correlation is indicated by * (*p* < 0.1), ** (*p* < 0.05), *** (*p* < 0.001). For families significantly correlated with the parameters evaluated, a linear regression (**B**) was obtained, demonstrating their relative abundances along the concentration gradient.

The correlation analysis between the microbiota at the taxonomic level of the family and the concentrations of SCFA (Figure 7A) was performed. As for acetic acid, contrary trends were observed for the Enterobacteriaceae and Veillonellaceae families; while the former has its abundance reduced in samples with higher concentrations of this acid, the latter showed a positive correlation in turn. The negative correlation between Enterobacteriaceae and acetate could be explained by the adverse effect of acetate towards bacteria from this family [121]. Higher SCFA and lower abundance of *Escherichia-Shigella* and *Klebsiella* were found after fermented milk containing probiotic strains (*Lb. paracasei* CNCM I-1518, *Lb. paracasei* CNCM I-3689, and *Lb. rhamnosus* CNCM I-3690) was administered for 28 days to patients under *Helicobacter pylori* eradication therapy [122].

Microorganisms from the *Veillonella* genus, belonging to the Veillonellaceae family, are unable to break down carbohydrates and use lactate for growth [123]; a higher abundance of the Veillonellaceae family in the gut microbiota of infants was associated with the administration of lactose [124] Therefore, *Veillonella* might have used lactate formed by microorganisms during milk fermentation or by microorganisms from the gut microbiota to produce acetate. Moreover, bacteria from the Veillonellaceae family are known for their ability to use intermediate or end products from the bacterial digestion of polysaccharides to produce acetate [125,126]. In our study, this family may probably have used substances from the breakdown of polysaccharides in the fruit pulp to produce acetate.

Butyric and propionic acids were correlated with two and one families, respectively. For butyric acid, Xanthomonadaceae and Sphingobacteriaceae presented a negative correlation with this SCFA. The correlation between these two families and butyrate has not been previously observed. At this point, it is unclear whether this significant correlation is influenced by other variables and is, therefore, causal. The reduction in Xanthomonadaceae and Sphingobacteriaceae may have been caused by other factors, such as competition for attachment sites and for nutrients, and/or production of antimicrobial substances. Nevertheless, the increase in butyric acid may also have been the result of the metabolism of other bacteria present in the microbiota.

Clostridiaceae showed a positive correlation with propionic acid. It is known that some species from the Clostridiaceae family, such as *Clostridium propionicum*, can produce propionic acid from lactate fermentation [127]. This same species ferments alanine, leading to the production of propionate via the pyruvate, lactate, and acrylate pathway [128]. Considering that milk has alanine in its aminoacidic profile, we hypothesize that *Clostridium* may have produced propionate through this route.

The correlation analysis between the microbiota at the taxonomic level of the family and the concentrations of ammonia pointed to a correlation with the abundance observed in nine families (Figure 7A), and the vast majority showed a positive relationship with the increase in its concentration. Three families with the highest general relative abundance in the studied microbiota (Supplementary Figure S4), Bacteroidaceae, Ruminococcaceae, and Enterobacteriaceae, were significantly correlated with ammonia. In this sense, there is a correlation between the decline in the abundance of Bacteroidaceae and the increase in ammonia concentrations. The inverse is true for the Ruminococcaceae and Enterobacteriaceae families, whose abundances positively follow the increase in ammonia concentration (Figure 7B). In addition, other less abundant families (Actinomycetaceae, Atopobiaceae, Caulobacteraceae, Enterococcaceae, Moraxellaceae, and Rhizobiaceae) showed positive correlations with ammonia.

There was a negative correlation between Bacteriodaceae and ammonia. Bacteroidetes belonging to the Bacteriodaceae family are considered the main bacteria that maintain a healthy state and homeostasis in the intestinal microbiota [129], although the species belonging to this family are known for their ability to metabolize amino acids [130], which are one of the precursors of ammonia. Ammonia has an inhibitory effect on *Bacteroidetes*, so the higher the concentration of ammonia in the environment, the smaller the population of Bacteroidaceae [131].

On the other hand, there was a positive correlation between Ruminococcaceae and Enterobacteriaceae and ammonia. Ruminococcaceae and Enterobacteriaceae, as well as Lachnospiraceae and Clostridiaceae, are commensal microorganisms with a significant role in the digestion of amino acids and proteins, thus, justifying their positive correlation with ammonia production [131,132,133].

Enterobacteriaceae, in addition to being indicators of intestinal dysbiosis, are also known to cause disease to the host when present in inadequate proportions [134]. The common cause of disease development is the compromise, erosion of the villi and damage to intestinal cells, leading to inflammatory responses and cytokine release in the intestinal tissue, thus, reducing its potential for nutrient absorption [133].

Among the SCFA, the number of families correlated with acetic, butyric, and propionic acid were five, two, and one, respectively (Figure 7A). As for acetic acid, contrary trends are observed for the Enterobacteriaceae and Veillonellaceae families; the former has its abundance reduced in samples with higher concentrations of this acid, while the latter has a positive correlation.

Administration of functional products containing probiotics and prebiotics, resulting in increased SCFA and decreased ammonia, has been demonstrated in the literature over the years [120,135,136]. It is known that dairy products can influence SCFA and ammonia production through microbiota modulation [4]. In some cases, some proteins can bind to other nutrients, such as sugar, making them less digestible, undermining the bacteria present in the intestinal microbiota that use proteolytic pathways [137]. These proteins can even be used due to the proteolysis process conducted by starter LAB, such as *Str. thermophilus,* directly influencing the availability of types of substrates in fermented products, such as peptides, which can influence microbial abundance and, consequently, the production of SCFA and ammonia [138].

Freire et al. [120] showed that fermented goat milk, with or without grape pomace extract, had a positive effect on the metabolism of the intestinal microbiota, increasing the production of SCFA and decreasing the concentration of ammonium. Rodrigues et al. [116] reported an increase in SCFA in SHIME^®^ using healthy human microbiota after administering ice cream containing *Lb. acidophilus* and *B. animalis* and a decrease in ammonia after administering a dietary supplement containing *Lb. acidophilus* and *B. animalis* (both after 7 days of treatment).

## 4. Conclusions

The viability of probiotic LAB in FM was ≥6 log CFU/mL and was affected by the addition of the fruit pulp. The Bacteroidetes phylum had greater abundance in the microbial communities associated with FM formulations, followed by Firmicutes. The *Phocaeicola* genus was dominant in the samples from the formulations with fruit pulp, which resulted in a lower bacterial diversity (Gini–Simpson) if compared to the FMC. The *Bifidobacterium* genus was related to all FM formulations, while the *Alistipes* genus was related to FMPF and FMBP formulations, and the *Lactobacillus* and *Lacticaseibacillus* genera were related to FMC. Furthermore, the FM with added fruit pulp showed conservation of the central taxon and its relationships in the genera co-occurrence networks. Regarding metabolites, there was a correlation between ammonia and the three most abundant families of the microbiota. All results showed that the administration of probiotic fermented milk with fruit pulp, especially FMB, boosted the beneficial effects observed in the intestinal microbiota of healthy humans, as well as increased the production of SCFA in SHIME^®^ and decreased ammonium ions, which could be related to the presence of bioactive compounds. Therefore, producing FM with fruit pulp can be considered a promising strategy for supplying probiotic FM with functional characteristics, even though additional studies should be carried out focusing on evaluating the sensory characteristics of the product, as well as the required shelf-life time to guarantee the desired beneficial effect to consumers.

## Figures and Tables

**Table 1:** Viability of lactic acid bacteria (Log CFU/mL) of FM assessed in SHIME^®^.

Log CFU/mL	*L. casei*	*L. plantarum*	*S. thermophilus*
FMC	9.95 ± 0.00 A	9.87 ± 0.04 A	3.45 ± 0.21 A
FMPF	9.59 ± 0.02 B	9.43 ± 0.09 B	3.74 ± 0.06 A
FMB	8.69 ± 0.01 C	9.56 ± 0.03 B	3.78 ± 0.00 A

FMC—Fermented milk control, without pulp; FMPF—Fermented milk with passion fruit pulp; FMB—Fermented milk with buriti pulp. Different capital letters in the same column denote a significant difference (*p* < 0.05) among fermented milk samples.

**Table 2:** Topological characteristics and centrality measures of the co-occurrence networks of the genera present in fermented milk.

Attribute	FM	FMPF	FMB
N. of nodes	21	15	16
N. of edges (+/−)	32 (30/2)	38 (21/17)	33 (24/9)
N. of modules (sizes)	3 (12, 7, 2)	3 (11, 2, 2)	2 (11, 5)
Modularity	0.42	0.101	0.298
Clustering coefficient	0.809	0.848	0.75
Mean degree	3.048	5.067	4.125
Max. degree	6 (ID: 91)	9 (IDs: 13, 40)	8 (ID: 40)
Mean betweenness	6.095	1.333	2.625
Max. betweenness	30 (IDs: 37, 63)	12 (ID: 13)	13.7 (ID: 40)
Main hubs	ID: 63	IDs: 40	ID: 40

**Table 3:** Short-chain fatty acid levels and ammonium ion (mmol/L) in the vessels corresponding to the ascending colon during the experimental period.

Periods	Acetic Acid	Propionic Acid	Butyric Acid	$NH_{+}^{4}$
Control	22.35 ± 2.26 ^D^	3.21 ± 0.24 ^B^	14.03 ± 1.66 ^B^	557.22 ± 9.74 ^A^
FMC	110.67 ± 16.44 ^A^	1.74 ± 0.79 ^B^	24.56 ± 0.00 ^A^	492.11 ± 34.36 ^B^
Washout FMC	47.05 ± 3.31 ^CD^	1.01 ± 0.30 ^B^	11.24 ± 0.81 ^BC^	506.56 ± 29.43 ^AB^
FMPF	125.66 ± 10.07 ^A^	1.15 ± 0.00 ^B^	15.96 ± 0.16 ^B^	284.89 ± 6.04 ^D^
Washout FMPF	52.65 ± 2.79 ^CD^	3.79 ± 1.33 ^B^	7.72 ± 0.08 ^C^	496.57 ± 7.98 ^B^
FMB	108.90 ± 5.06 ^AB^	18.47 ± 3.32 ^A^	25.80 ± 1.87 ^A^	378.67 ± 6.15 ^C^
Washout FMB	71.04 ± 2.37 ^BC^	3.13 ± 0.98 ^B^	16.97 ± 0.79 ^B^	472.11 ± 6.27 ^B^

FMC—Fermented milk control without pulp, FMPF—Fermented milk with passion fruit pulp, FMB—Fermented milk with buriti pulp. Different capital letters in the same column denote a significant difference (*p* < 0.05) during the experimental periods.

**Table 4:** Short chain fatty acid levels and ammonium ion (mmol/L) in the vessels corresponding to the ascending colon during the experimental period.

Periods	Acetic Acid	Propionic Acid	Butyric Acid	$NH_{+}^{4}$
Control	22.35 ± 2.26 ^D^	3.21 ± 0.24 ^B^	14.03 ± 1.66 ^B^	557.22 ± 9.74 ^A^
FMC	110.67 ± 16.44 ^A^	1.74 ± 0.79 ^B^	24.56 ± 0.00 ^A^	492.11 ± 34.36 ^B^
Washout FMC	47.05 ± 3.31 ^CD^	1.01 ± 0.30 ^B^	11.24 ± 0.81 ^BC^	506.56 ± 29.43 ^AB^
FMPF	125.66 ± 10.07 ^A^	1.15 ± 0.00 ^B^	15.96 ± 0.16 ^B^	284.89 ± 6.04 ^D^
Washout FMPF	52.65 ± 2.79 ^CD^	3.79 ± 1.33 ^B^	7.72 ± 0.08 ^C^	496.57 ± 7.98 ^B^
FMB	108.90 ± 5.06 ^AB^	18.47 ± 3.32 ^A^	25.80 ± 1.87 ^A^	378.67 ± 6.15 ^C^
Washout FMB	71.04 ± 2.37 ^BC^	3.13 ± 0.98 ^B^	16.97 ± 0.79 ^B^	472.11 ± 6.27 ^B^

FMC—Fermented milk control, without pulp, FMPF—Fermented milk with passion fruit pulp, FMB—Fermented milk with buriti pulp. Different capital letters in the same column denote a significant difference (*p* < 0.05) during the experimental periods.

## Data Availability

All data generated by the current project are available upon request.

## Supplementary Materials

The following supporting information can be downloaded at: https://www.mdpi.com/article/10.3390/foods11244113/s1, Figure S1. Observation of ASVs in relation to the number of sampled sequences. The stabilization of the curves indicates the decrease in finding new sequence variants. CTRL: control; FM: fermented milk without fruit pulp; WFM: washout of fermented milk without fruit pulp; FMPF: fermented milk with passion fruit pulp; WFMPF: washout of fermented milk with passion fruit pulp; FMB: fermented milk with buriti pulp; WFMB: washout of fermented milk with buriti pulp; Figure S2. Relative abundance of communities at the phyla level associated with fermented milk without the addition of fruit pulp (A) and with passion fruit pulp (B) or buriti pulp (C) addition. CTRL: control; FM: fermented milk without fruit pulp; WFM: washout of fermented milk without fruit pulp; FMPF: fermented milk with passion fruit pulp; WFMPF: washout of fermented milk with passion fruit pulp; FMB: fermented milk with buriti pulp; WFMB: washout of fermented milk with buriti pulp; Figure S3. Differential abundant (DA) taxa among FM formulations against their control, as follows: control period vs. FMC (A), washout of FMC vs. FMPF (B) and washout of FMPF vs. FMB (C). The dotplots represent the mean intensity of the difference (fold-change) complemented by the standard error. The taxa are preceded by letters, that which represent the taxonomic level, namely, Phylum (p), Class (c), Order (o), Family (f), Genus (g), and Species (s); Figure S4. Relative abundance of communities at the family level associated with fermented milk without the addition of fruit pulp (A) and with passion fruit pulp (B) or buriti pulp (C) addition. CTRL: control; FM: fermented milk without fruit pulp; WFM: washout of fermented milk without fruit pulp; FMPF: fermented milk with passion fruit pulp; WFMPF: washout of fermented milk with passion fruit pulp; FMB: fermented milk with buriti pulp; WFMB: washout of fermented milk with buriti pulp.
