# Supplementary material for: Functional Fermented Milk with Fruit Pulp Modulates the In Vitro Intestinal Microbiota
Source: Foods. 2022 Dec 19;11(24):4113. doi: 10.3390/foods11244113 (PMC9778618; doi:10.3390/foods11244113)
Supplement: Supplementary file 1 [file foods-11-04113-s001.zip › foods-2063008-supplementary.pdf]

Supplementary Figures

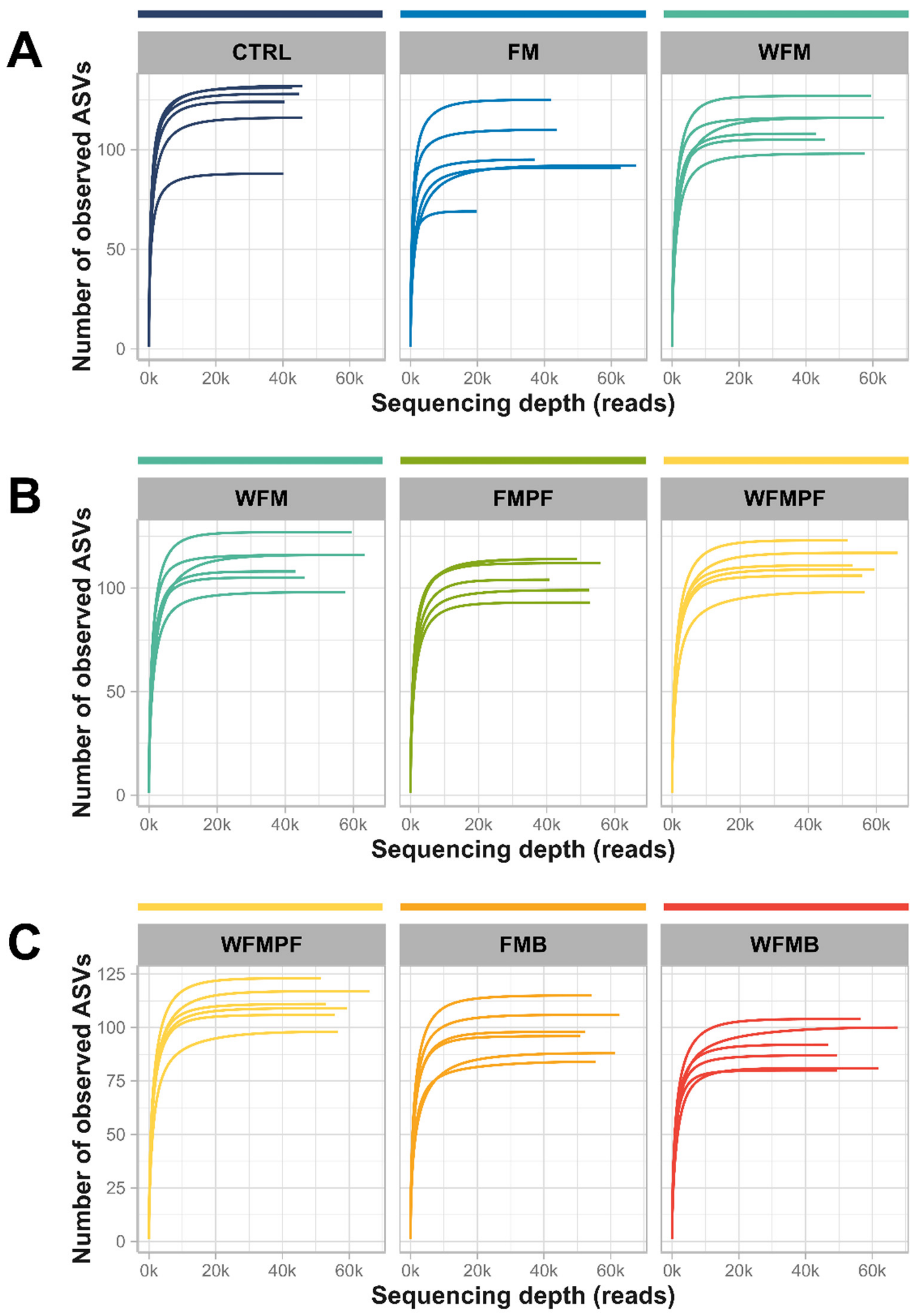

Figure S1. Observation of ASVs in relation to the number of sampled sequences. The stabilization of the curves indicates the decrease in finding new sequence variants. CTRL: control; FM: fermented milk without fruit pulp; WFM: washout of fermented milk without fruit pulp; FMPF: fermented milk with passion fruit pulp; WFMPF: washout of fermented milk with passion fruit pulp; FMB: fermented milk with buriti pulp; WFMB: washout of fermented milk with buriti pulp.

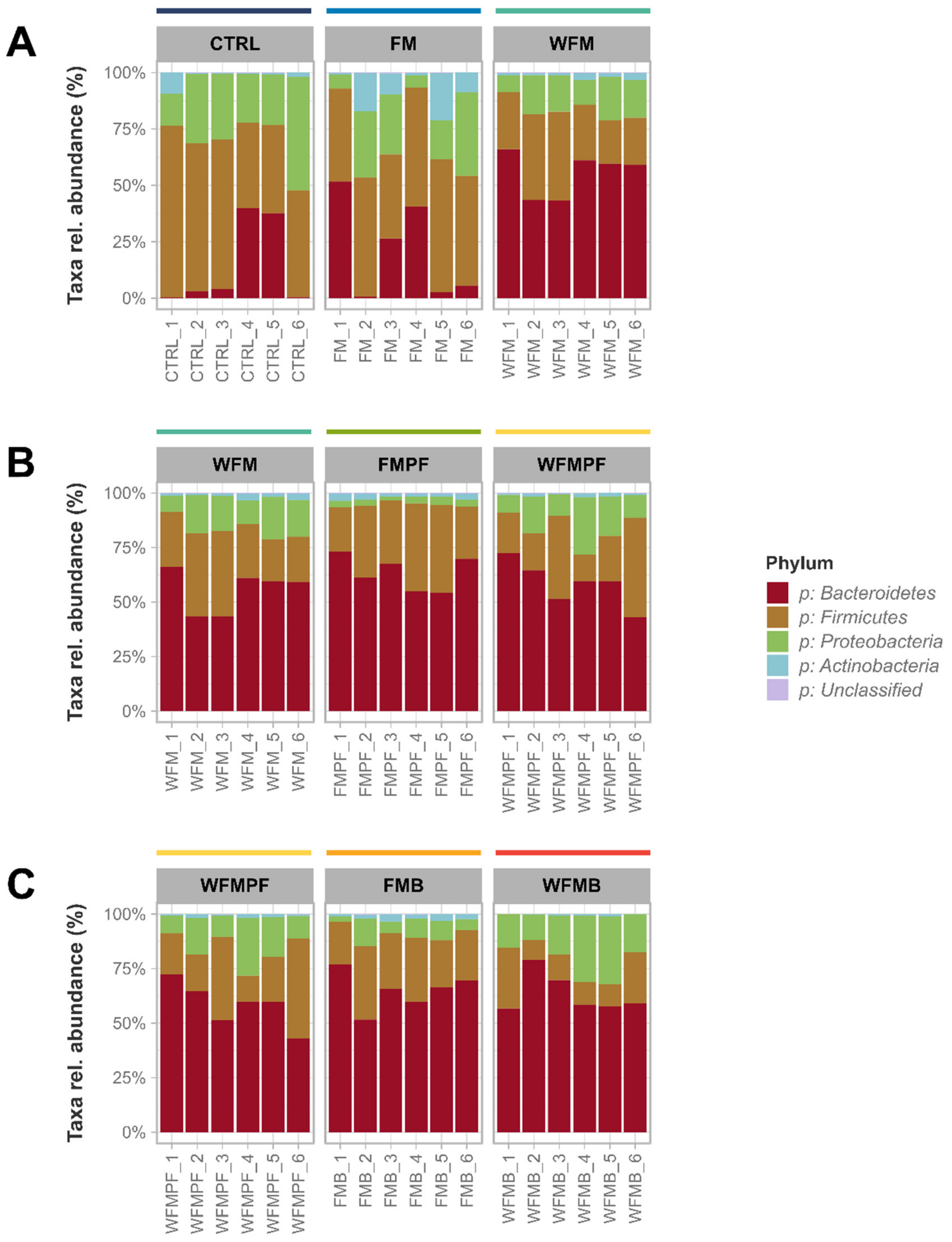

Figure S2. Relative abundance of communities at the phyla level associated with fermented milk without the addition of fruit pulp (A) and with passion fruit pulp (B) or buriti pulp (C) addition. CTRL: control; FM: fermented milk without fruit pulp; WFM: washout of fermented milk without fruit pulp; FMPF: fermented milk with passion fruit pulp; WFMPF: washout of fermented milk with passion fruit pulp; FMB: fermented milk with buriti pulp; WFMB: washout of fermented milk with buriti pulp.

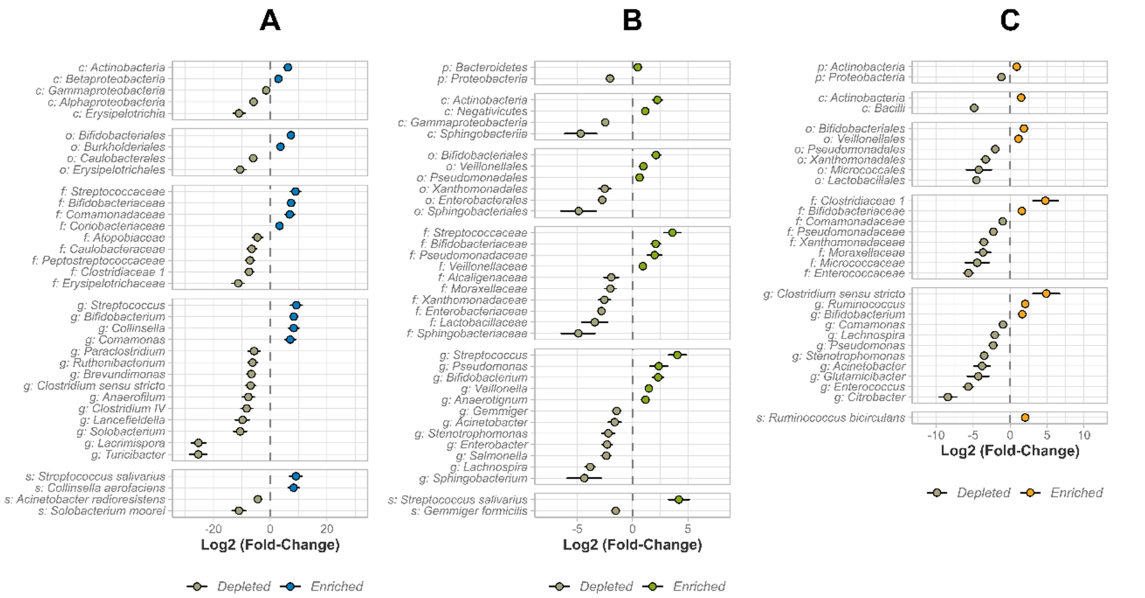

Figure S3. Differential abundant (DA) taxa among FM formulations against their control, as follows: control period vs. FMC (A), washout of FMC vs. FMPF (B) and washout of FMPF vs. FMB (C). The dotplots represent the mean intensity of the difference (fold-change) complemented by the standard error. The taxa are preceded by letters, that which represent the taxonomic level, namely, Phylum (p), Class (c), Order (o), Family (f), Genus (g), and Species (s).

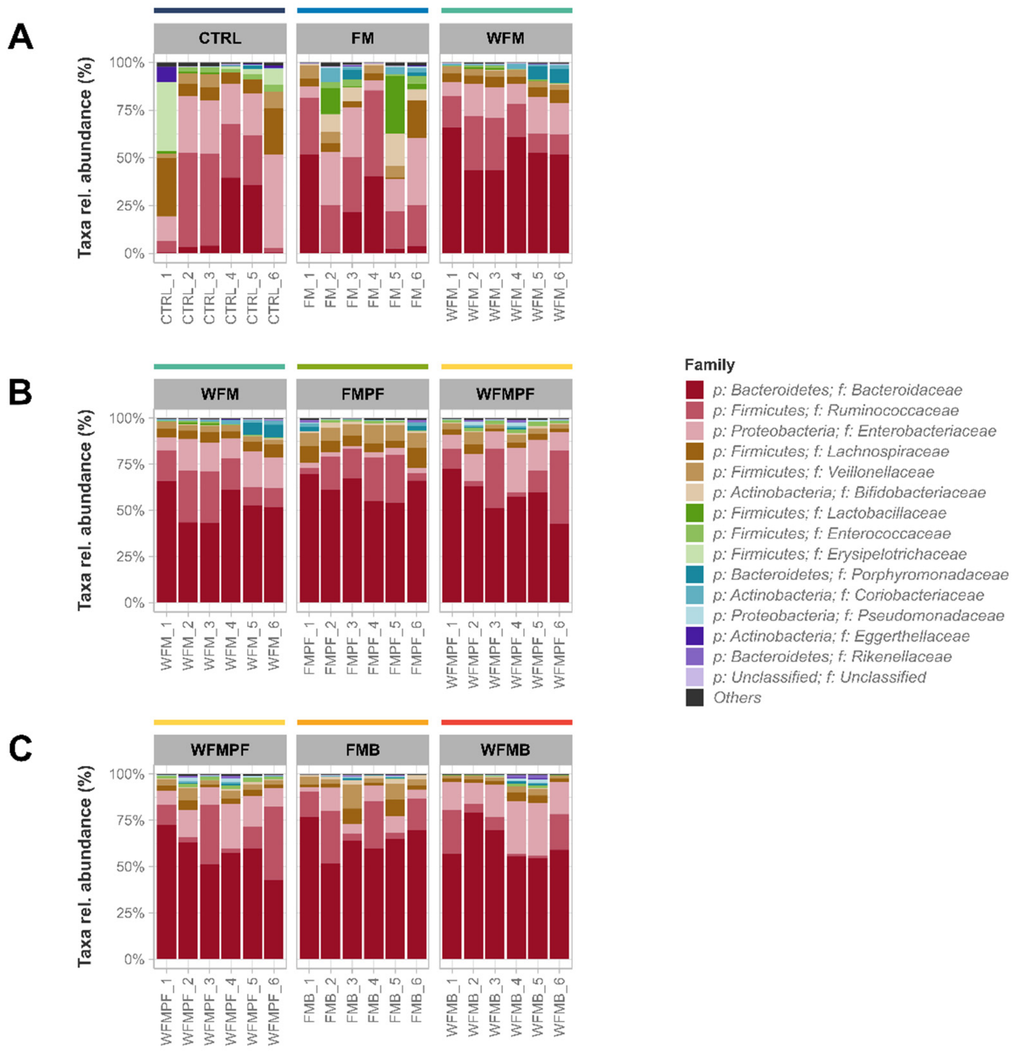

Figure S4. Relative abundance of communities at the family level associated with fermented milk without the addition of fruit pulp (A) and with passion fruit pulp (B) or buriti pulp (C) addition. CTRL: control; FM: fermented milk without fruit pulp; WFM: washout of fermented milk without fruit pulp; FMPF: fermented milk with passion fruit pulp; WFMPF: washout of fermented milk with passion fruit pulp; FMB: fermented milk with buriti pulp; WFMB: washout of fermented milk with buriti pulp.
